# A lipasin/Angptl8 monoclonal antibody lowers mouse serum triglycerides involving increased postprandial activity of the cardiac lipoprotein lipase

**DOI:** 10.1038/srep18502

**Published:** 2015-12-21

**Authors:** Zhiyao Fu, Abdul B. Abou-Samra, Ren Zhang

**Affiliations:** 1 Center for Molecular Medicine and Genetics, School of Medicine, Wayne State University, 540 East Canfield Street, Detroit, MI 48201, USA; 2 Division of Endocrinology, School of Medicine, Wayne State University, Detroit, MI 48201, USA; 3 Department of Medicine, Hamad Medical Corporation, Doha, Qatar

## Abstract

Lipasin/Angptl8 is a feeding-induced hepatokine that regulates triglyceride (TAG) metabolism; its therapeutical potential, mechanism of action, and relation to the lipoprotein lipase (LPL), however, remain elusive. We generated five monoclonal lipasin antibodies, among which one lowered the serum TAG level when injected into mice, and the epitope was determined to be EIQVEE. Lipasin-deficient mice exhibited elevated postprandial activity of LPL in the heart and skeletal muscle, but not in white adipose tissue (WAT), suggesting that lipasin suppresses the activity of LPL specifically in cardiac and skeletal muscles. Consistently, mice injected with the effective antibody or with lipasin deficiency had increased postprandial cardiac LPL activity and lower TAG levels only in the fed state. These results suggest that lipasin acts, at least in part, in an endocrine manner. We propose the following model: feeding induces lipasin, activating the lipasin-Angptl3 pathway, which inhibits LPL in cardiac and skeletal muscles to direct circulating TAG to WAT for storage; conversely, fasting induces Angptl4, which inhibits LPL in WAT to direct circulating TAG to cardiac and skeletal muscles for oxidation. This model suggests a general mechanism by which TAG trafficking is coordinated by lipasin, Angptl3 and Angptl4 at different nutritional statuses.

Patients with type 2 diabetes are often associated with hypertriglyceridemia, which is an independent risk factor for cardiovascular disease^1^,^2^,^3^. The lipoprotein lipase (LPL), which hydrolyzes triglycerides (TAG) in lipoproteins, plays a critical role in determining plasma TAG levels, and therefore, its activity is tightly controlled to meet the needs of various tissues under different nutritional statuses and physiopathological conditions^4^,^5^,^6^,^7^. An effective way to appreciate the tissue-specific regulation of LPL activity is to gain an understanding of the fasting-fed cycle. During fasting, LPL activity is upregulated in the heart and skeletal muscle^8^,^9^,^10^,^11^,^12^,^13^, which, in turn, take up fatty acids for energy production. In the fed state, LPL activity is upregulated in white adipose tissue^5^,^14^,^15^,^16^, which, in turn, takes up fatty acids for storage. However, the molecular mechanism by which LPL partitions fatty acids among these tissues during the fasting-fed cycle is still incompletely understood.

It has been well established that Angptl3 and Angptl4 are critical regulators of LPL activity^17^,^18^,^19^,^20^,^21^. Angptl3 inhibits LPL activity, and consistently, Angptl3 overexpression or deletion increases or lowers serum TAG levels, respectively^17^,^18^. Angptl4 was identified as a PPARγ target gene induced by fasting in adipocytes^22^,^23^,^24^. Angptl4 increases plasma TAG levels also by inhibiting LPL activity^25^. Consistently, Angptl4-null mice have lower plasma TAG levels and increased post-heparin plasma LPL activity, while overexpression of Angptl4 increases plasma TAG levels and decreases post-heparin plasma LPL activity^18^. Both Angptl3 and Angptl4 need to be proteolytically cleaved to release the N-terminal functional domain to inhibit LPL activity^26^,^27^,^28^,^29^,^30^. Consistently, injection of monoclonal antibodies against N-terminal domains of Angptl4 or Angptl3, mimics phenotypes of Angptl4- or Angptl3-null mice^31^,^32^. Sequence variations of both ANGPTL3 and ANGPTL4 have been linked to human lipid profiles by various genome-wide association studies (GWAS)^19^,^33^,^34^,^35^.

Recently, much focus has been placed on a previously uncharacterized gene, officially named C19ORF80 (human) and Gm6484 (mouse) according to the HUGO Gene Nomenclature Committee^36^. Here the gene is referred to as lipasin^37^,^38^,^39^, despite various names being used in literatures, such as RIFL^40^, Angptl8^41^ and betatrophin^42^. Lipasin is highly enriched in the liver and adipose tissues, including both white and brown adipose tissues^38^,^40^,^41^. Fasting reduces expression of lipasin and feeding dramatically induces its expression^38^,^40^,^41^. Overexpression of lipasin in the mouse liver using adenovirus dramatically increases serum TAG levels^38^,^41^; conversely, mice deficient in lipasin have reduced TAG levels^43^,^44^. Therefore, both loss- and gain-of-function studies on mice indicate that lipasin is a critical regulator of TAG metabolism. Multiple studies have identified C19ORF80 sequence variations that are associated with lipid profiles in human GWAS^41^,^45^,^46^,^47^,^48^. It has been shown that circulating lipasin levels in humans are elevated in both type 1^49^,^50^ and type 2 diabetes^51^,^52^,^53^ in various populations. Taken together, lipasin is clearly a nutritionally-regulated liver-enriched circulating factor that regulates TAG metabolism.

To further study the function of lipasin, its therapeutical potential and mechanism of action, we asked the following questions: 1) Can lipasin-neutralizing antibodies reduce serum TAG levels, and if so, what is the mechanism? 2) How is the nutritional regulation of lipasin related to its function? That is, why is lipasin strongly induced by feeding to regulate TAG metabolism? Here, we show that lipasin negatively regulates LPL activity specifically in the heart and skeletal muscle, and that a lipasin monoclonal antibody lowers serum TAG levels by up-regulating postprandial cardiac LPL activity. Based on these results, we propose a model by which TAG trafficking is coordinated by lipasin, Angptl3 and Angptl4 at different nutritional statuses.

## Results

### Generation of monoclonal lipasin antibodies

To examine whether lipasin neutralization lowers serum TAG levels, we generated five monoclonal lipasin antibodies, as described in the Methods section. Briefly, a mouse lipasin recombinant protein^38^ was used as an immunogen to immunize BALB/c mice. Antibody titers were monitored by ELISA, and once high titers were achieved, splenocytes were harvested and fused with myeloma cells. In total, 5 hybridoma cell lines were obtained and following hybridomas expansion and antibody purification, we obtained about 30 mg of each of the purified monoclonal antibodies. To confirm the binding specificity, we performed both western blotting analysis and ELISA. All the 5 antibodies, AB-1 to AB-5, recognized recombinant lipasin protein by western blotting analysis (not shown). ELISA with lipasin in different dilutions showed corresponding binding signals (Suppl. Table 1 for AB-2).

### A lipasin monoclonal antibody lowers serum triglycerides in mice

**Figure 1:**
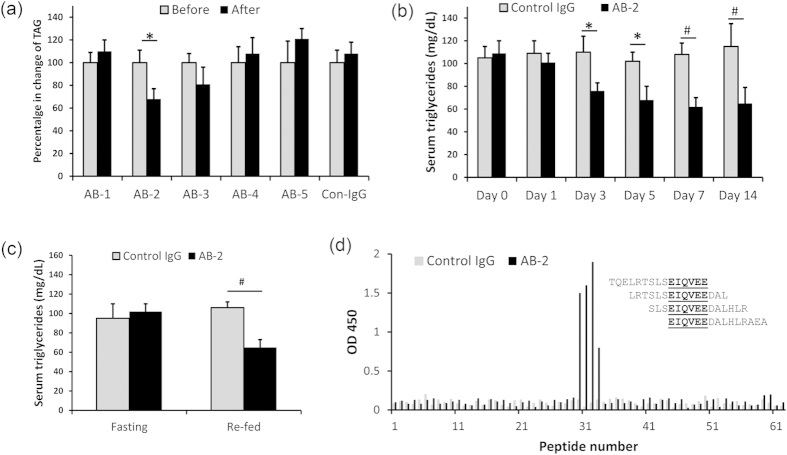
A lipasin monoclonal antibody lowers serum triglycerides in mice. (**a**) Using a recombinant lipasin as an antigen, 5 monoclonal antibodies were generated in mice using ImmunoPlus^TM^ technology. The antibodies (30 mg/kg body weight) were injected *i.p.* daily at 9 AM. Blood samples were collected on day 3 at 10 AM. One (AB-2) of the five lipasin monoclonal antibodies lowers serum triglycerides in mice. (**b**) Injection of the antibody AB-2 lowers serum triglyceride levels. AB-2 was injected *i.p.* daily and blood samples were collected before injection (day 0), and on days 1, 3, 5, 7 and 14. (**c**) AB-2 lowers serum triglyceride levels 4 hours following refeeding, but not at the fasting state. (**d**) Epitope mapping shows that AB-2 binds to segment EIQVEE of the lipasin protein. Sixty two overlapping peptides spanning the entire lipasin protein were synthesized. ELISA was performed to examine the binding of these peptides with AB-2. All mice were male, 6–8 weeks old. Mice were fasted for 24 hours, or followed by refeeding for 4 hours. There were 5 mice in each experimental condition. Data are expressed as mean ± sem. *P < 0.05; ^#^P < 0.01.

Each of the 5 antibodies or a control IgG from non-immunized mice was injected *i.p.* into wild-type mice (30 mg/kg body weight). Because lipasin level is sensitive to food intake, antibody injection and blood collection were performed at specific times of the day. Unless otherwise indicated, the antibodies were injected once a day at 9 AM, and blood samples were collected at 10 AM daily. On day 3, among the 5 antibodies, only AB-2 significantly lowered serum TAG levels with about a 30% decrease (Fig. 1a).

To further examine the effects of AB-2, we monitored the serum TAG changes for two weeks in mice with AB-2 injection. Staring on day 3, mice receiving AB-2 had significantly lower levels of serum TAG. On day 3, serum TAG levels in mice with control IgG and AB-2 injection were 110 ± 14 and 76 ± 7 (mg/dL, P < 0.05) respectively. It appeared that after day 5, the magnitude of reduction in TAG levels became stabilized. TAG levels in control mice and mice with AB-2 injection on day 5 were 102 ± 8 and 68 ± 12 (mg/dL, P < 0.05), respectively; on day 7 they were 108 ± 10 and 62 ± 8 (mg/dL, P < 0.01), respectively; on day 14, they were 115 ± 20 and 65 ± 14 (mg/dL, P < 0.01), respectively. That is, on days 5, 7, and 14, reductions of TAG were 33%, 43%, and 43%, respectively (Fig. 1b). Therefore, AB-2 is a lipasin monoclonal antibody that lowers mouse TAG levels starting as early as day 3 following daily injection, and the maximum reduction in TAG levels was around 40% at the fed state.

Because lipasin is a nutritionally-regulated gene, that is, its expression is reduced by fasting, and dramatically increased by feeding, we examined the effect of AB-2 in mice during the fasting-refed cycle. Mice received AB-2 injections daily (at 9 AM), and were divided into 2 groups, with both groups being fasted for 24 hours starting at 10 AM on day 7. On day 8, one group of mice was re-fed for 4 hours before blood collection. Under fasting, TAG levels in control and AB-2 injected mice were comparable; following refeeding, TAG levels in AB-2 injected mice became lower, 106 ± 6 *vs.* 65 ± 9 (mg/dL, P < 0.01) (Fig. 1c). Therefore, AB-2 injection lowered the serum TAG levels only in re-fed mice, but not in fasted mice.

### Epitope mapping of AB-2

We performed epitope mapping to examine the binding site of AB-2 in the lipasin protein. A peptide library was generated containing 62 15-amino-acid long peptides with 12 amino-acid overlapping (Suppl. Fig. 1). All cysteines in the peptides were replaced with serines and the peptides were synthesized with N-terminal biotinylation. ELISA was performed with plates pre-coated with streptavidin. We found that peptides 30, 31, 32 and 33 showed strong binding signals for AB-2. To confirm the binding affinity, we performed ELISA using peptides from 28 to 35, with different dilutions of the peptides, and the peptides 30-33 showed linearly decreasing signals with higher dilutions (Suppl. Table 2). The overlapping segment in the four peptides was EIQVEE, and therefore the binding site of AB-2 was EIQVEE, which contains amino acids from 97 to 102 in the mouse lipasin protein (Fig. 1d). An Angptl4 monoclonal antibody showed a TAG-lowering effect, and the epitope, the SE1 region, was in Angptl4 amino acids from 29–53^31^,^32^. The location of the epitope of AB-2 is quite different from the SE1 region, suggesting different mechanisms of action for the antibody AB-2.

### Serum triglyceride levels of lipasin KO and overexpressing mice during the fasting-refed cycle

**Figure 2:**
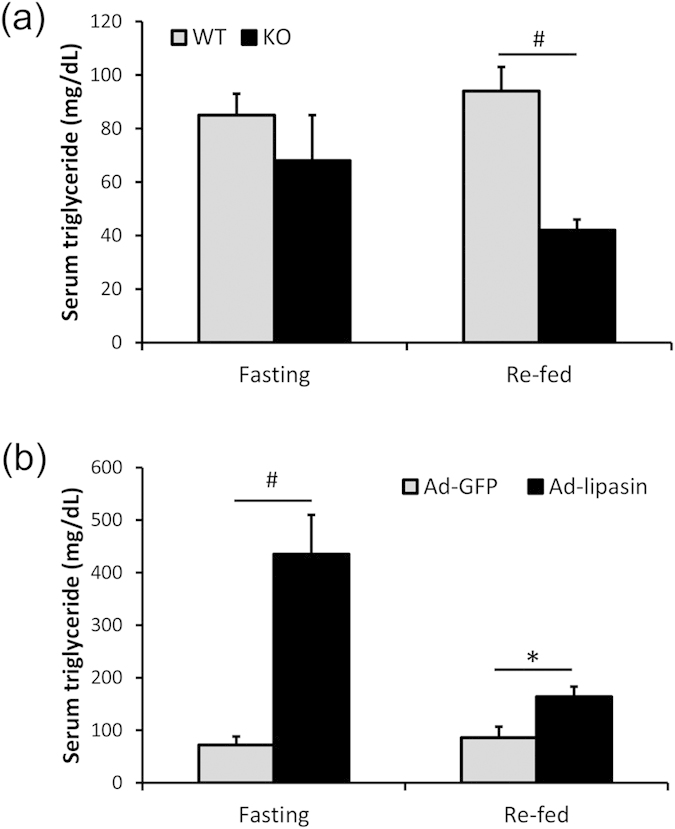
Serum triglyceride levels of lipasin KO and overexpressing mice during the fasting-refed cycle. (**a**) Serum triglyceride levels were lower in the KO mice only at the re-fed stage. (**b**) Adenovirus-mediated expression of lipasin increased mouse serum triglycerides. Each mouse was injected with 5 × 10^8^ pfu (diluted in 200 μl of saline) of adenoviruses expressing either green fluorescent protein (GFP) or lipasin. Serum triglyceride levels were determined in blood samples collected 8 days after adenovirus infection with fasting started on day 7. Mice were either fasted for 24 hours or re-fed for 4 hours following the 24-hour fasting. (n = 6–8/group). Data are expressed as mean ± sem. *P < 0.05; ^#^P < 0.01.

Because fasting and feeding profoundly affect how lipasin regulates TAG metabolism, we compared and contrasted the phenotypes of lipasin-deficient and lipasin overexpressing mice during the fasting-refed cycle. Under 24-hour fasting, no significant difference in TAG levels was observed between WT and lipasin KO littermates. But 4 hours after refeeding, serum TAG levels were reduced by about 50% (P < 0.01) in the lipasin KO mice (Fig. 2a).

Next, we overexpressed a recombinant lipasin using adenovirus-lipasin by tail vein injection. Seven days after virus infection, mice were fasted for 24 hours or followed by a 4-hour refeeding. In fasted mice, lipasin overexpression dramatically increased serum TAG levels. The TAG level in lipasin-overexpressing mice was more than 6-fold higher than that in control mice receiving adeno-GFP (P < 0.01). In re-fed mice, lipasin overexpression still significantly increased serum TAG levels, but with much less magnitude (about 2-fold higher) (Fig. 2b). Therefore, lipasin deficiency lowers TAG levels only in fed mice, and the increase in the serum TAG level by lipasin overexpression was much dramatic in the fasting state than in the fed state.

### Lipasin deficiency increases postprandial activity of lipoprotein lipase in cardiac and skeletal muscles

Fasting and refeeding have a profound impact on how lipasin regulates TAG levels, as shown in mice with lipasin antibody injection (Fig. 1c), deletion (Fig. 2a) and overexpression (Fig. 2b). LPL is a critical factor in TAG metabolism, and LPL is highly regulated in a tissue-specific manner. We therefore examined how tissue heparin-releasable LPL activity is changed during the fasting-refed cycle in WT and lipasin KO mice.

**Figure 3:**
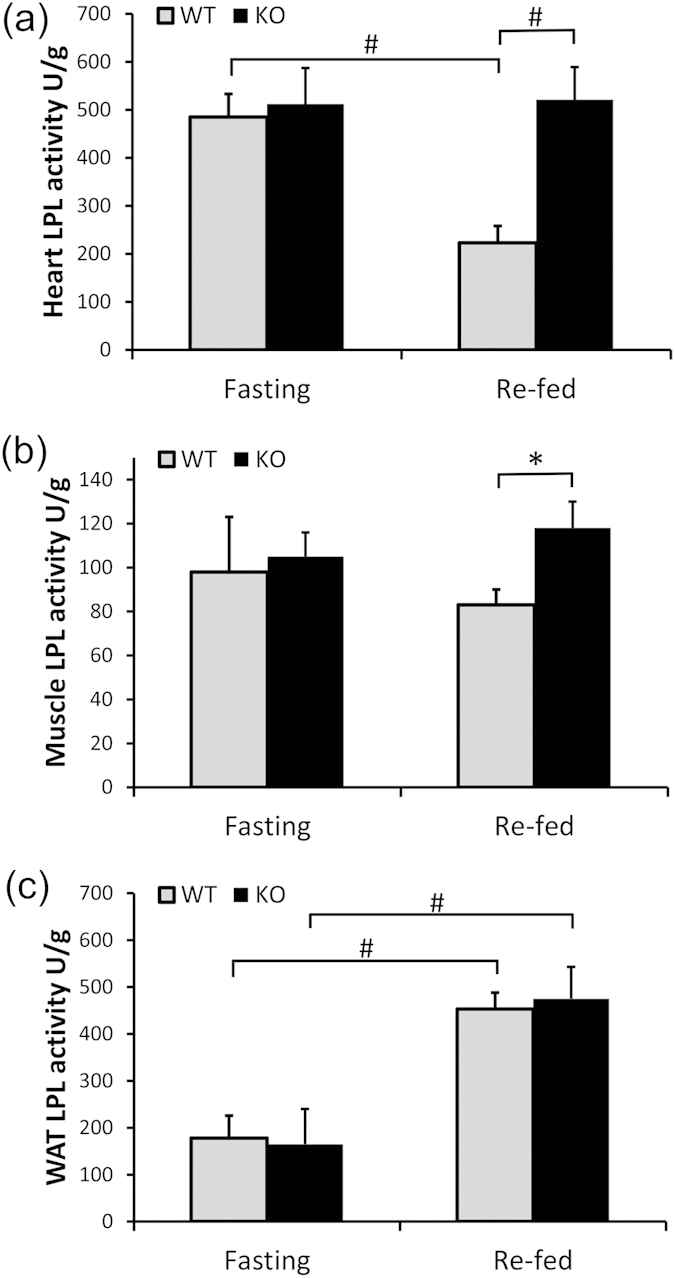
Increased postprandial activity of lipoprotein lipase in the heart and skeletal muscle. Heparin-releasable LPL activity was measured in (**a**) heart, (**b**) muscle and (**c**) white adipose tissue of both wild type and lipasin KO mice under fasting or re-fed conditions. LPL activity was expressed as unit per gram of tissue. One unit is defined as one nmol fatty acid generation per min at 37°C. Mice were either fasted for 24 hours or re-fed for 4 hours following the 24-hour fasting. (n = 4–6/group). Data are expressed as mean ± sem. *P < 0.05; ^#^P < 0.01.

In the heart, heparin-releasable LPL activity fell after refeeding from 485 ± 48 to 223 ± 35 (U/g, P < 0.01). But in the KO mice, the postprandial decrease in heparin-releasable LPL activity in the heart was totally abolished (512 ± 75 *vs.* 521 ± 68 U/g, under fasting and refed, respectively). That is, at the fed state, the KO mice had a more than 2-fold increase in postprandial cardiac heparin-releasable LPL activity (P < 0.01, Fig. 3a). In skeletal muscle of WT mice, we did not observe a significant difference in LPL activity, but following refeeding, KO mice had higher LPL activity than WT mice (P < 0.05, Fig. 3b).

In WAT, feeding induced a dramatic increase in heparin-releasable LPL activity in both WT and KO mice. In the WT mice, the activities at fasting and refed were 178 ± 48 *vs.* 453 ± 35 U/g, P < 0.01, and in the KO mice, 165 ± 75 *vs.* 475 ± 68 U/g, P < 0.01). That is, in both WT and KO mice, there was a close to 3-fold increase in postprandial LPL activity. But in striking contrast to the heart and skeletal muscle, KO and WT mice showed comparable WAT LPL activity (Fig. 3c). Therefore, lipasin negatively regulates LPL activity specifically in cardiac and skeletal muscles.

### Activity of cardiac LPL in mice with the antibody AB-2 injection or lipasin overexpression

Lipasin negatively regulates the activity of heparin releasable LPL in cardiac and skeletal muscles at the fed state, and therefore, it is possible that in mice with antibody injection or adenovirus infection, the phenotype in TAG metabolism is caused by changes in LPL activity in these tissues. To test this hypothesis, we examined tissue LPL activity in mice with either antibody injection or adenovirus-lipasin infection.

**Figure 4:**
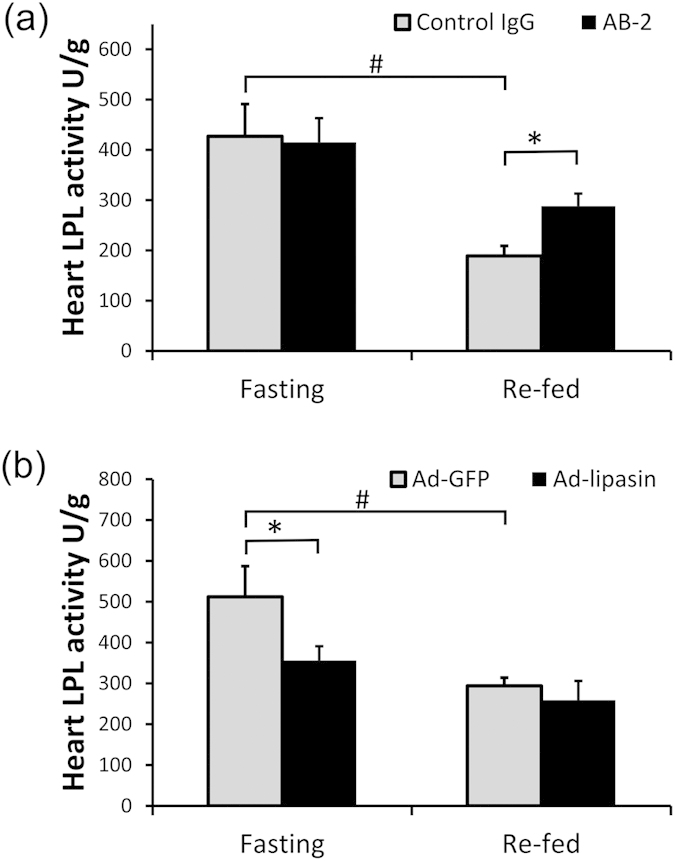
Injection of the antibody AB-2 increases postprandial activity of the cardiac lipoprotein lipase. Heparin-releasable LPL activity was measured in heart in (**a**) lipasin KO mice and wild-type controls, and (**b**) mice infected with adenoviruses overexpressing either GFP or lipasin. Refer to the Method section for the timing of fasting, amount and timing for virus injection. LPL activity was expressed as unit per gram of tissue. One unit is defined as one nmol fatty acid generation per min at 37°C. Mice were either fasted for 24 hours or re-fed for 4 hours following the 24-hour fasting. (n = 4–5/group). Data are expressed as mean ± sem. *P < 0.05; ^#^P < 0.01.

The antibody AB-2 or control IgG was *i.p.* injected daily for 7 days. In the heart, as expected, refeeding reduced heparin-releasable LPL activity (P < 0.01) in mice with control IgG injection. In the mice with AB-2 injection, the LPL activity was significantly increased at the fed state, compared with the control mice (P < 0.05, Fig. 4a), and at the fasting state, there was no significant difference. Consistently, in mice with adenovirus-lipasin injection, compared to the GFP control mice, mice showed significant reduction in cardiac LPL activity at the fasting state (P < 0.05, Fig. 4b). At the re-fed state, LPL activity was comparable between control and lipasin-overexpressing mice.

## Discussion

**Figure 5:**
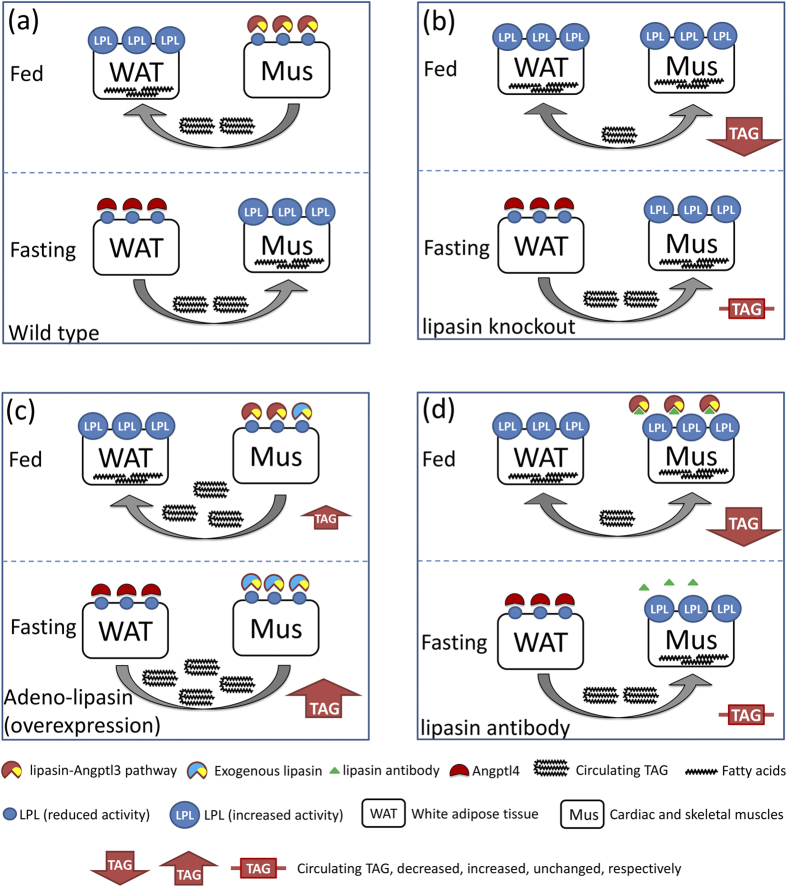
A model of triglyceride metabolism regulated by lipasin, Angptl3 and Angptl4. Lipasin negatively regulates the activity of LPL in cardiac and skeletal muscles, likely through an Angptl3-dependent mechanism. Food intake dramatically induces the expression of lipasin in the liver, which, in turn, secretes lipasin into the circulation; while food intake suppresses the expression of Angptl4, an LPL inhibitor in WAT. Conversely, fasting dramatically induces Angptl4, but suppresses the expression of lipasin. Triglyceride trafficking is coordinated by the lipasin-Angptl3 pathway and Angptl4 (**a**) under normal physiology, (**b**) in lipasin-deficient mice, (**c**) in lipasin-overexpressing mice, and (**d**) in mice with lipasin antibody injection. Refer to Discussion for details. AB, antibody, Ad, adenovirus; KO, knockout; LPL, lipoprotein lipase; Mus, cardiac and skeletal muscles; TAG, triglyceride; WAT, white adipose tissue.

In lipasin KO mice, heparin-releasable postprandial LPL activity was increased in the heart and skeletal muscle, but not in WAT. The above results suggest that lipasin inhibits LPL specifically in cardiac and skeletal muscles. Consistently, TAG levels in the lipasin KO mice were lower in the fed state, but not in the fasting state; conversely, lipasin-overexpressing mice showed dramatically or modestly increased TAG levels in the fasting or fed states, respectively. We therefore propose the following Angptl3-4-8 model (Fig. 5), in which lipasin, Angptl3 and Angptl4 regulate TAG metabolism and energy fuel trafficking between muscles and WAT at different nutritional states.

It has been well established that lipasin is dramatically reduced by fasting and induced by feeding^38^,^39^,^40^,^41^, and that Angptl4 is a fasting-induced LPL inhibitor in WAT^15^,^16^. Under normal physiology, following food intake, increased lipasin inhibits LPL activity, likely through an Angplt3-dependent mechanism, in cardiac and skeletal muscles to suppress their ability to hydrolyze circulating TAG. At the same time, reduced Angptl4 releases LPL activity in WAT, to hydrolyze TAG and thereby take up more fatty acids into WAT. Conversely, under fasting, increased Angptl4 inhibits WAT LPL activity, and at the same time, reduced lipasin releases LPL activity in cardiac and skeletal muscles, which, in turn, take up more fatty acids (Fig. 5a).

Angptl3 by itself is not nutritionally regulated^54^, but its activity can be potentially nutritionally-regulated by lipasin. One possibility is that lipasin activates Angptl3 by enhancing its cleavage, releasing its N-terminal domain, which in turn inhibits LPL in cardiac and skeletal muscles^41^. It is also possible that lipasin and Angptl3 form a complex that travels to these tissues to inhibit LPL. The two possibilities are distinct, in that according to the former, lipasin does not need to be physically located in the heart endothelium, while the latter implies that lipasin has to be functional by interacting with heart endothelium LPL. Therefore, in this model, we denote the effect of lipasin by the lipasin-Angptl3 pathway. Both lipasin and Angptl3 should be in the same functional pathway, because Angptl3-null mice show consistent changes with lipasin-null mice in terms of LPL activity in the heart and skeletal muscles^55^. But the difference is that Angptl3-null mice also exhibited higher LPL activity in WAT^55^, while in lipasin KO mice, LPL activity in WAT was unaltered. Lipasin is highly induced during adipose differentiation and highly induced by feeding in WAT^40^, and therefore, current results support a notion that lipasin in WAT has LPL-independent functions.

In the lipasin KO mice, at fed state, a lack of lipasin fails to inhibit LPL in cardiac and skeletal muscles, thereby hydrolyzing more circulating TAG, resulting in a phenotype of low serum TAG levels. But under fasting, both wild type and lipasin KO mice have diminished lipasin, and therefore show similar TAG levels (Fig. 5b). In mice with lipasin overexpression with adenovirus tail-vein injection, the phenotype of increased serum TAG levels is more dramatic at the fasting state, because LPLs in both WAT and muscles are inhibited by Angptl4 and exogenous lipasin, respectively (Fig. 5c). In mice with lipasin neutralizing antibody injection, in the fed state, lipasin activity is blocked by the antibody, resulting in higher LPL activity in cardiac and skeletal muscles, and therefore lower serum TAG levels. But under fasting, serum TAG levels are similar between mice with or without antibody injection, because of a low level of lipasin in both experimental groups of mice (Fig. 5d).

Olivecrona and coworkers found that a transcription-dependent mechanism modulates heart LPL activity^56^. In rats injected with actinomycin D, which blocks transcription, LPL activity in the heart was increased. Therefore, they proposed that food intake induces a protein, which, in turn, inhibits postprandial cardiac LPL activity^56^. Lipasin is highly likely to be the feeding-induced protein that inhibits postprandial LPL activity in the above study.

Lipasin is highly enriched in liver and adipose tissues, and can be secreted from the liver into the circulation. In WAT and BAT, where lipasin is expressed, it is still uncertain whether lipasin acts in an endocrine, autocrine or paracrine manner. But here we show that lipasin KO mice have elevated LPL activity in the heart and skeletal muscles, in which lipasin is not expressed. Therefore, lipasin functions, at least in part, in an endocrine manner.

Patients with type 2 diabetes are often associated with diabetic dyslipidemia, characterized by increased levels of plasma TAG, decreased plasma HDL-C and postprandial lipemia^57^,^58^. It is generally believed that circulating lipasin levels are increased in type 2 diabetes. For instance, in mice lipasin mRNA levels are highly induced by obesity and insulin resistance^38^,^40^,^42^. In humans, circulating lipasin levels have been shown to be increased in type 2 diabetes in various populations^50^,^51^,^52^,^53^,^59^,^60^. The model presented here suggests a mechanism in which lipasin plays a role in diabetic dyslipidemia. Increased lipasin in diabetes inhibits LPL in cardiac and skeletal muscles, hydrolyzing less circulating TAG in TAG-rich lipoproteins, resulting in higher plasma TAG levels. However, the validity of this model in type 2 diabetes needs to be examined by future studies.

The model is simplified in the sense that an organism has different nutritional statuses beyond being fasted and fed. It is likely that the different nutritional statuses change the relative abundance and balance between Angptl4 and lipasin to fine-tune the partition of TAG among tissues. The Angptl3-4-8 model, however, not only explains the TAG phenotypes in mice with altered lipasin levels, but also suggests a general mechanism by which TAG is partitioned among cardiac and skeletal muscles *vs.* WAT under different nutritional statuses.

## Methods

### Antibody generation

A recombinant lipasin was generated as previously described^38^, and the protein was used as an immunogen to immunize 5 BALB/c mice, and were boosted every two weeks, in collaboration with GenScript using the ImmunoPlus^TM^ technology (GenScript, Piscataway, NJ). Serum titers were monitored by ELISA, and once high titers were achieved, splenocytes were harvested from the immunized mice and fused with myeloma cells. In total, 5 hybridoma cell lines derived from 5 parental clones were obtained. To scale up, the 5 hybridomas were expanded using the standard roller bottle method, followed by antibody purification by Protein G affinity columns, resulting in a yield of about 30 mg for each antibody. Both western blotting analysis and ELISA were performed to confirm the binding specificity of the 5 antibodies.

### Epitope mapping

We generated a peptide library that contained 62 15-amino-acid long peptides with 12 amino-acid overlapping. All cysteines in the peptides were replaced with serines and the peptides were synthesized with N-terminal biotinylation (GenScript). ELISA was then performed to determine the peptides that bind to the AB-2 antibody. Refer to Supplemental Materials for detailed procedure of ELISA on epitope mapping.

### Mice

Mice were housed at 22–24 °C with a 14-h light, 10-h dark cycle and provided with *ad libitum* water and a chow diet (6% calories from fat, 8664; Harlan Teklad, Indianapolis, IN) unless otherwise indicated. Lipasin knockout mice (B6;129S5-Gm6484^tm1Lex^/Mmucd) were obtained from the Mutant Mouse Resource Research Centers^43^. All fasting experiments were performed by fasting mice for 24 hours starting at 10 AM, and refeeding refers to 4 hours of refeeding following the 24-hour fasting, unless indicated otherwise.

For the antibody screening experiment, each group had 5 mice. The lipasin antibodies (30 mg/kg body weight) or control IgG (purified IgG from non-immunized mice, GenScript) were injected *i.p.* daily at 9 AM. Blood samples were collected on day 3 at 10 AM. In experiments to examine the antibody AB-2 effects, mice received AB-2 injections daily (at 9 AM), mice were divided into 2 groups, and both groups of mice were fasted for 24 hours starting at 10 AM on day 7. Then one group of mice was re-fed for 4 hours before blood collection.

Adenovirus injection was as previously described^38^. Mice were injected into the tail vein with 5 × 10^8^ pfu (diluted in 200 μl of saline) of adenoviruses expressing GFP or lipasin. To examine the TAG level changes during fasting-refed cycle, mice with overexpression of lipasin or GFP were divided into two groups with 5 mice in each group. All mice were fasted, with the 24-hour fasting started on day 7, at 10 AM, and in the refeeding group, blood collection was performed 4 hours after refeeding. In experiments to examine postprandial LPL activity, AB-2 antibody was *i.p.* injected daily for 8 days at 9 AM. The 24-hour fasting started on day 7 at 10 AM. Tissues were collected after fasting for 24 hours or 4 hours refeeding following the fasting. For mice with adenovirus infection, each mouse was injected with 5 × 10^8^ pfu (diluted in 200 μl of saline) of adenoviruses expressing either GFP or lipasin, and the tissue was collected 8 days following virus infection. The 24-hour fasting started on day 7 following virus infections. All animal experiments were performed in accordance with the protocols approved by the Animal Care and Use Committee of Wayne State University.

### LPL activity

Tissues including the heart, muscle (soleus), and white adipose tissue (epididymal fat) were isolated from anesthetized mice. Tissues weighing about 100 mg were minced into pieces (about 1 mm^3^) in cold Krebs-Ringer-phosphate buffer (KRP), pH 7.4 and then were incubated in a shaking 37°C water bath for 45 min in 0.5 ml of KRP with 15 μg/ml heparin. Tissue lysates were then centrifuged at 10,000 g for 10 min, and supernatants were collected for LPL activity assay, using the LPL activity assay kit (Biovision, Milpitas, CA) according to manufacturer instructions. LPL activity is expressed as unit per gram of tissue, with 1 unit defined as the amount of 1 nmol of fatty acid generation per min at pH7.4 at 37°C. Serum TAG levels were determined by the triglyceride quantification kit (Biovision).

### Statistical analysis

Data are expressed as the mean ± sem. Statistical significance was tested with unpaired two-tailed Student’s *t* tests unless otherwise indicated. The differences were considered statistically significant if P < 0.05.

## Additional Information

**How to cite this article**: Fu, Z. *et al.* A lipasin/Angptl8 monoclonal antibody lowers mouse serum triglycerides involving increased postprandial activity of the cardiac lipoprotein lipase. *Sci. Rep.* **5**, 18502; doi: 10.1038/srep18502 (2015).

## Supplementary Material

Supplementary Information
