# Supplementary material for: A lipasin/Angptl8 monoclonal antibody lowers mouse serum triglycerides involving increased postprandial activity of the cardiac lipoprotein lipase
Source: Sci Rep. 2015 Dec 21;5:18502. doi: 10.1038/srep18502 (PMC4685196; doi:10.1038/srep18502)
Supplement: Supplementary Information [file srep18502-s1.pdf]

A lipasin/Angptl8 monoclonal antibody lowers mouse serum triglycerides involving increased postprandial activity of the cardiac lipoprotein lipase

Zhiyao Fu, Abdul B. Abou-Samra and Ren Zhang

Supplemental Materials

Supplementary Figure 1. The peptide library for epitope mapping

1	MAVLALSLLWTLASA	22	GHSLGLYDRALEFLG	43	RDTVRRLQVQLRGAW
2	LALSLLWTLASAVRP	23	LGLYDRALEFLGTEV	44	VRRLQVQLRGAWLGQ
3	SLLWTLASAVRPAPV	24	YDRALEFLGTEVRQG	45	LQVQLRGAWLGQAHQ
4	WTLASAVRPAPVAPL	25	ALEFLGTEVRQGQDA	46	QLRGAWLGQAHQEFE
5	ASAVRPAPVAPLGGP	26	FLGTEVRQGQDATQE	47	GAWLGQAHQEFETLK
6	VRPAPVAPLGGPEPA	27	TEVRQGQDATQELRT	48	LGQAHQEFETLKARA
7	APVAPLGGPEPAQYE	28	RQGQDATQELRTSLS	49	AHQEFETLKARADKQ
8	APLGGPEPAQYEELT	29	QDATQELRTSLSEIQ	50	EFETLKARADKQSHL
9	GGPEPAQYEELTLLF	30	TQELRTSLSEIQVEE	51	TLKARADKQSHLLWA
10	EPAQYEELTLLFHGA	31	LRTSLSEIQVEEDAL	52	ARADKQSHLLWALTG
11	QYEELTLLFHGALQL	32	SLSEIQVEEDALHLR	53	DKQSHLLWALTGHVQ
12	ELTLLFHGALQLGQA	33	EIQVEEDALHLRAEA	54	SHLLWALTGHVQRQQ
13	LLFHGALQLGQALNG	34	VEEDALHLRAEATAR	55	LWALTGHVQRQQREM
14	HGALQLGQALNGVYR	35	DALHLRAEATARSLG	56	LTGHVQRQQREMAEQ
15	LQLGQALNGVYRATE	36	HLRAEATARSLGEVA	57	HVQRQQREMAEQQQW
16	GQALNGVYRATEARL	37	AEATARSLGEVARAQ	58	RQQREMAEQQQWLRQ
17	LNGVYRATEARLTEA	38	TARSLGEVARAQQAL	59	REMAEQQQWLRQIQQ
18	VYRATEARLTEAGHS	39	SLGEVARAQQALRDT	60	AEQQQWLRQIQQRLH
19	ATEARLTEAGHSLGL	40	EVARAQQALRDTVRR	61	QQWLRQIQQRLHTAA
20	ARLTEAGHSLGLYDR	41	RAQQALRDTVRRLQV	62	LRQIQQRLHTAALPA
21	TEAGHSLGLYDRALE	42	QALRDTVRRLQVQLR		

Supplementary Figure 1

A peptide library was generated containing 62 15-amino-acid long peptide with 12 amino-acid overlapping. All cysteines in the peptides were replaced with serines and the peptides were synthesized with N-terminal biotinylation.

Supplementary table 1. ELISA for recombinant lipasin using the monoclonal antibody AB-2

	Dilution	AB-2 (A450nm)
1	1:1,000	1.437
2	1:3,000	1.436
3	9,000	1.409
4	27,000	1.327
5	81,000	1.180
	Blank	0.085
The titer: >1:81,000		

Starting dilution: 1:1,000 (equivalent to 1µg/ml)

The titer is the highest dilution with Positive/Black > 2.1.

ELISA antigen: recombinant lipasin

Coating: 1 µg/ml, 100 µl/well

Coating buffer: phosphate buffered saline, pH 7.4

Secondary antibody: Goat anti-mouse IgG (H+L), HRP conjudated.

Supplementary Table 2. ELISA verification of the AB-2 binding peptides

	28	29	**30**	**31**	**32**	**33**	34	35
AB-2 0.5 μg/ml	0.055	0.105	**1.177**	**1.148**	**1.057**	**0.729**	0.093	0.06
AB-2 -0.1 μg/ml	0.056	0.069	**0.915**	**0.851**	**0.441**	**0.238**	0.063	0.056
AB-2 0.02 μg/ml	0.057	0.058	**0.574**	**0.544**	**0.183**	0.082	0.054	0.053
ControlAb-0.5 μg/ml	0.059	0.063	0.060	0.059	0.061	0.060	0.060	0.059
ControlAb-0.1 μg/ml	0.054	0.058	0.057	0.056	0.056	0.056	0.057	0.055
ControlAb-0.02 μg/ml	0.052	0.056	0.054	0.057	0.054	0.055	0.055	0.058
1×PBS	0.058	0.053	0.052	0.050	0.051	0.052	0.051	0.050
